# Whole-body thermal therapies for symptom management and well-being in people assigned female at birth with chronic pelvic pain, dysmenorrhoea, endometriosis or adenomyosis: a scoping review protocol

**DOI:** 10.1136/bmjopen-2026-120614

**Published:** 2026-08-03

**Authors:** Laura Elizabeth Cowley, Mayara S Bianchim, Kate Isherwood, Judith Roberts, April Rees, Rachel Joseph, Rebecca Anthony

**Affiliations:** 1 Population Data Science, Swansea University, Swansea, UK; 2 School of Psychology and Women’s Health Research Wales Centre, Cardiff University, Cardiff, UK; 3 Centre for Population Health, Swansea University, Swansea, UK; 4 School of Sport and Health Sciences, Cardiff Metropolitan University, Cardiff, UK; 5 Department of Psychology, Aberystwyth University, Aberystwyth, UK; 6 Biomedical Sciences, Swansea University, Swansea, UK; 7 School of Psychology, Cardiff University, Cardiff, UK; 8 DECIPHer, Cardiff University, Cardiff, UK

**Keywords:** Dysmenorrhea, Pelvic Pain, COMPLEMENTARY MEDICINE, GYNAECOLOGY, Review

## Abstract

**Abstract:**

**Introduction:**

Dysmenorrhoea is one of the most frequent manifestations of chronic pelvic pain. Endometriosis and adenomyosis are among the most common pathological causes of secondary dysmenorrhoea. Symptoms often persist despite treatment, so individuals frequently engage in self-management strategies. One self-management approach that may have potential benefits for chronic pain is whole-body thermal therapy, which involves exposure to heat or cold stimuli inducing systemic physiological responses. The aim of this scoping review is to map and synthesise the available literature on the use and reported benefits of whole-body thermal therapies in individuals assigned female at birth (AFAB) with chronic pelvic pain, dysmenorrhoea, endometriosis and adenomyosis.

**Methods and analysis:**

The proposed scoping review will adhere to the PRISMA-ScR (Preferred Reporting Items for Systematic reviews and Meta-Analysis, Extension for Scoping Reviews) checklist. We will search the Embase, Scopus, Medline, American Psychological Association (APA) PsycINFO, Web of Science and CINAHL Ultimate electronic databases for primary studies published in English from the year 2000. All articles will be independently assessed by two reviewers for eligibility. Data will be synthesised using framework synthesis and findings will be presented descriptively in tabular and narrative format.

**Ethics and dissemination:**

The proposed study does not require ethical approval as it is a scoping review of the existing literature and will only describe and synthesise previously published data. We will consult with an established patient and public involvement (PPI) panel who will assist in interpreting and disseminating the findings. We will produce and disseminate creative outputs (eg, visual illustrations) that capture discussions during PPI panel meetings. Findings will be disseminated in a peer-reviewed journal, at conferences and via webinars with relevant networks.

**Trial registration number:**

This protocol has been registered as a preprint on the Open Science Framework platform: https://osf.io/zxp3j.

**STRENGTHS AND LIMITATIONS OF THIS STUDY**

- The review will be conducted systematically; two reviewers will independently conduct study screening, selection and data extraction, ensuring methodological rigour and the production of a high-quality review.
- The review will be informed by an established panel of patient and public involvement contributors, and adhere to recommended reporting standards, in accordance with the PRISMA-ScR (Preferred Reporting Items for Systematic reviews and Meta-Analysis, Extension for Scoping Reviews) checklist.
- We will search a wide range of organisational websites to identify grey literature sources.
- In line with scoping review guidance, we will not conduct a quality appraisal of the included studies or formal risk of bias assessment.
- The number of studies included may be insufficient to draw robust conclusions.

## Introduction

### Background

 Chronic pelvic pain (CPP) is a common and debilitating condition defined as persistent or recurrent pain in the lower abdomen or pelvis lasting for at least 3 to 6 months. It is estimated to affect approximately 15%–25% of individuals of reproductive age and represents a major cause of reduced quality of life and healthcare utilisation.^1^ Dysmenorrhoea, or painful menstruation, is one of the most frequent manifestations of pelvic pain, with the prevalence of severe dysmenorrhoea ranging from 2% to 29%.^2^ Dysmenorrhoea, especially when it is severe, may significantly impair daily functioning and well-being and has been shown to have an impact on performance and attendance at school or work, and engagement with social and sporting opportunities.^3^ It can be classified as primary, occurring in the absence of identifiable pelvic pathology, or secondary, with menstrual pain arising from an underlying condition. Among the most common pathological causes of secondary dysmenorrhoea are endometriosis and adenomyosis.

Endometriosis is a chronic inflammatory condition characterised by the presence of endometrial-like tissue outside the uterus, most commonly on the pelvic organs and tissues.^4^ It is estimated to affect approximately 10% of women of reproductive age worldwide, representing a substantial global health burden.^5^ Adenomyosis involves the presence of endometrial-like tissue and stroma within the myometrium^6^ and the global prevalence in the general population is estimated to be 1%.^7^ These conditions frequently coexist and share overlapping symptom profiles, including severe dysmenorrhoea and chronic pelvic pain, reflecting shared inflammatory and neurogenic mechanisms of pain.^5 7^ Consequently, interventions targeting severe dysmenorrhoea and the underlying conditions of endometriosis and adenomyosis are central to the management of chronic pelvic pain.

Systematic reviews have reported that chronic pelvic pain, severe dysmenorrhoea, endometriosis and adenomyosis are associated with a high prevalence of psychiatric comorbidities, including anxiety and depression.^8–11^ From an economic perspective, the cost of absenteeism due to severe dysmenorrhoea and heavy periods alongside endometriosis, fibroids and ovarian cysts in the UK is estimated to be nearly £11 billion per annum.^12^ Despite their prevalence and impact, there is currently no cure for endometriosis or adenomyosis, and treatment strategies are primarily aimed at managing symptoms and improving quality of life. Available management options include pharmacological therapies such as non-steroidal anti-inflammatory drugs and hormonal treatments, as well as surgical interventions to remove endometriotic lesions or adenomyotic tissue.^13^ However, these treatments are not universally effective, and recurrence or persistence of symptoms is common. Many individuals continue to experience dysmenorrhoea and chronic pelvic pain despite medical or surgical treatment, and some may be unable to tolerate available therapies due to side effects or contraindications. Therefore, individuals with chronic pelvic pain and dysmenorrhoea frequently engage in self-management strategies to cope with ongoing symptoms, including behavioural, lifestyle and non-pharmacological approaches such as localised heat therapy, rest, dietary changes and exercise.^14^

One self-management approach that may have potential benefits for chronic pelvic pain is whole-body thermal therapy, which involves exposure to heat or cold stimuli that induce systemic physiological responses.^15^ Thermal therapies, including sauna bathing, hot water immersion and cold-water exposure, are increasingly used in both clinical and non-clinical settings for symptom management and recovery.^15^ Evidence from other pain and musculoskeletal conditions suggests that thermal exposure may influence pain perception and symptom relief through several mechanisms. Heat exposure has been shown to promote muscle relaxation, increase blood flow and reduce pain sensitivity, whereas cold exposure may reduce inflammation, tissue swelling and nociceptive signalling.^15 16^ In addition to these local effects, whole-body thermal stimuli may elicit broader cardiovascular and neurophysiological responses, including changes in circulation, autonomic nervous system activity and endogenous pain modulation.^17^ Given their low cost, accessibility and non-invasive nature, whole-body thermal therapies may represent a feasible self-management strategy for individuals experiencing chronic pelvic pain or dysmenorrhoea. Evidence supporting the use of localised heat therapy for menstrual pain is relatively well established, with a recent systematic review suggesting that topical heat application can provide pain relief comparable to non-steroidal anti-inflammatory drugs in some cases.^18^ However, the evidence regarding the potential benefits of whole-body thermal therapies—such as sauna bathing or ice baths—for dysmenorrhoea and chronic pelvic pain remains limited and less well characterised.

### Context and rationale

The need for this review was identified following the 2022 Women’s Health in Wales Discovery Report,^19^ in which over 3800 women across Wales highlighted gynaecological conditions and menstrual health as key priorities for research and healthcare. These findings informed the Welsh Government’s 2025 Women’s Health Plan for Wales,^20^ a 10-year strategy that identifies menstrual health as the most urgent area for action.

In response, the EMPOWER (Endometriosis Management and Promotion of Well-being using Evidence-based Research) Women’s Health Research Network was established. This interdisciplinary network brings together researchers, clinicians, policymakers, third sector organisations and individuals with lived experience to advance research into endometriosis and related conditions. The network is supported by a patient and public involvement panel (EMPOWER-VOICES) and a multidisciplinary steering group (EMPOWER-CONNECT), ensuring integration of clinical, research and lived experience perspectives. A key priority for the EMPOWER Network is to evaluate the evidence for whole-body thermal therapies, as potential self-management strategies. This focus reflects recognised gaps in the evidence base. Previous evidence mapping has identified a lack of primary research on interventions that support symptom management and improve quality of life for individuals with endometriosis.^21^ In addition, priority-setting exercises by the James Lind Alliance^22^ and the National Institute for Health and Care Excellence (NICE)^23^ have highlighted the need for effective non-surgical, non-pharmacological and self-management approaches to manage pain, fatigue and psychological well-being. Notably, limited evidence currently exists to inform which information and support interventions are effective in improving quality of life in this population.^21^

Given these gaps, a comprehensive examination of the existing evidence on thermal therapies is warranted to evaluate their potential as accessible, low-cost interventions for symptom management across related conditions including chronic pelvic pain, severe dysmenorrhoea, endometriosis and adenomyosis. We conducted preliminary searches of the Cochrane Database of Systematic Reviews and PROSPERO in January 2026 to determine if any previous systematic reviews had been conducted or protocols submitted which aimed to summarise existing evidence on the use of whole-body thermal therapies for the management of these conditions. We found three completed systematic reviews on heat therapy or non-pharmacological interventions for primary dysmenorrhoea^18 24 25^; we will check these for relevant primary studies as part of our review process. We found a further four ongoing (unpublished) systematic reviews on self-management strategies, heat therapy and cold-based therapies for dysmenorrhoea or menstrual cycle symptoms, and heat therapy for chronic pain management. There were no reviews on heat/cold therapy for endometriosis or adenomyosis, and no reviews specifically focusing on whole-body thermal therapies for any of the conditions.

The aim of this scoping review is to map and synthesise the available literature on the use and reported benefits of whole-body thermal therapies in individuals assigned female at birth (AFAB) with chronic pelvic pain, dysmenorrhoea, endometriosis and adenomyosis. Specifically, this review seeks to describe the extent and characteristics of the existing literature, identify evidence gaps and inform future research and clinical practice.

### Review question

What evidence exists on the use and effect of whole-body thermal therapies for symptom management and well-being in people AFAB with dysmenorrhoea, unexplained chronic pelvic pain, endometriosis (or symptoms indicative of endometriosis), or adenomyosis?

## Methods and analysis

This protocol was developed in accordance with best practice guidance following the Preferred Reporting Items for Systematic Review and Meta-Analysis Protocols (PRISMA-P) checklist^26^ (online supplemental appendix 1). In addition, this protocol has been registered and made publicly available on the Open Science Framework: https://osf.io/zxp3j.

In line with recent methodological guidance,^27 28^ the proposed scoping review will follow a nine-step process, with the first three steps undertaken during protocol development:

1. Defining and aligning the objective/s and question/s.
2. Developing and aligning the inclusion criteria with the objective/s and question/s.
3. Describing the planned approach to evidence searching, selection, extraction, analysis and presentation.
4. Searching for the evidence.
5. Selecting the evidence.
6. Extracting the evidence.
7. Analysis of the results.
8. Presentation of the results.
9. Summarising the evidence in relation to the purpose of the review, making conclusions and noting any implications of the findings.

The purpose of a scoping review is to ascertain the nature and extent of the existing literature on a given topic, enabling a broad overview of available evidence and highlighting knowledge gaps. Unlike systematic reviews, scoping reviews do not require a formal appraisal of the quality of included studies.^29^ Despite this, scoping reviews are recognised as a robust and systematic approach to evidence synthesis. We will begin the review in February 2026 and complete it by September 2026. The review will be reported using the PRISMA-ScR (Preferred Reporting Items for Systematic reviews and Meta-Analysis, Extension for Scoping Reviews) checklist.^30^ This will ensure that the review process is documented as clearly and rigorously as possible.

### Patient and public involvement (PPI)

Five members of the review team have lived experience of endometriosis or adenomyosis. In addition, the review will be informed by up to 10 patient and public involvement (PPI) contributors on the EMPOWER-VOICES panel with lived experience of confirmed or suspected endometriosis. PPI contributors will take part in either an in-person workshop or online discussion and will have the opportunity to review the research question, search strategy, and data extraction form and contribute to the interpretation of the emerging findings. Their input will help contextualise results and support the interpretation of gaps in the evidence where relevant. During the in-person workshop, we will use creative and participatory methods to capture, reflect on and share emerging discussions, through collaboration with a creative organisation specialising in arts-based knowledge exchange and inclusive public engagement. This inclusive and participatory approach will ensure that those with lived experience are not only consulted but are actively involved in shaping the research process. All PPI activities will be informed by the UK Standards for Public Involvement,^31^ with all panel members receiving appropriate support and financial reimbursement. PPI activities will be reported based on the Guidance for Reporting Involvement of Patients and the Public Checklist^32^ and impact recorded using the Public Involvement in Research Impact Toolkit (PIRIT).^33^ It is worth emphasising that some aspects of the review detailed below may be further developed based on the PPI input (the research questions, eligibility criteria or search strategy).

### Eligibility criteria

Any published, peer-reviewed, primary research studies will be considered. Theses, dissertations and conference abstracts will be excluded. Studies will be included from all geographical areas but must be published in English for feasibility and due to a lack of resources for translation of articles. The search will be restricted to studies published from 2000 to 2026 to enhance the feasibility of the review and ensure relevance to contemporary clinical practice and research standards. Diagnostic approaches, management pathways and delivery of non-pharmacological therapies for endometriosis and related conditions have evolved substantially over the past two decades.^34^ Additionally, studies published after 2000 are more likely to employ standardised outcome measures and reporting frameworks, improving comparability and interpretability of findings. We followed the SPIDER (Sample, Phenomenon of Interest, Design, Evaluation, Research type) framework, which has been found to be particularly useful for conducting mixed-methods reviews,^35^ to structure the research question, eligibility criteria, search strategy and data extraction form (table 1).

**Table 1:** Eligibility criteria for the articles to be included in the scoping review, following the SPIDER framework

Framework component	Inclusion criteria	Exclusion criteria
Sample	People AFAB with dysmenorrhoea, unexplained chronic pelvic pain, endometriosis (or symptoms indicative of endometriosis, eg, bowel or urinary symptoms reported alongside dysmenorrhoea, cyclical pain), or adenomyosis.	Studies not focused on any of these conditions, studies focused on people assigned male at birth, animal studies.
Phenomenon of interest	Use and effect of whole-body thermal therapies, delivered in any setting. Whole-body thermal therapies are defined as whole-body heat therapy, whole-body cold therapy and contrast therapy, delivered through modalities such as baths, immersion, showers or saunas. Where studies investigate multiple forms of thermal therapies, these will be reviewed and information extracted on whole-body therapies only, if available.	Localised thermal therapies (eg, hot water bottles, heating pads or ice packs) will be excluded. Studies using heat/cold for surgical therapy (eg, thermo/cryo-ablation) will be excluded.
Design	Randomised controlled trials, quasi-experimental studies, focus groups, intervention studies, interview studies, survey studies, case studies or observational studies.	N/A we aim to include a wide range of study designs.
Evaluation	Symptom management (including but not limited to pain, bloating, fatigue, bowel or bladder symptoms), and physical and mental well-being (including but not limited to quality of life, sleep quality, energy, anxiety, mood).	N/A we aim to include a wide range of possible symptoms and well-being outcomes.
Research type	We will include quantitative, qualitative or mixed-methods studies. Clinical guidelines will be included if they contain information/evidence about the use and effect of whole-body thermal therapies for the included conditions.	Literature reviews, scoping reviews, and systematic reviews themselves will be excluded but we will screen and include the primary studies from these where relevant. Letters, commentaries, editorials, book chapters and study protocols will be excluded.

AFAB, assigned female at birth; SPIDER, Sample, Phenomenon of Interest, Design, Evaluation, Research type.

### Information sources

To identify potentially eligible articles, we will search six electronic databases from 2000 to February 2026. We will search the Embase, Web of Science and Scopus databases, and search the Medline, American Psychological Association (APA) PsycINFO and CINAHL Ultimate databases via the EBSCO platform. We will conduct forward and backward citation searching of included articles and relevant reviews using the citationchaser R Package and Shiny App,^36^ to identify articles the database searches may have missed. In addition, we will search the websites of the following organisations for grey literature: WHO, United Nations Children’s Fund (UNICEF), United Nations Population Fund (UNFPA), National Institute for Clinical Excellence (NICE), Endometriosis UK, Welsh Government, Welsh Parliament (Senedd), National Health Service (NHS) Wales, NHS England, the Menstrual Health Project, well-being of Women, the Endometriosis Foundation, European Society of Human Reproduction and Embryology (ESHRE) and The Royal College of Obstetricians and Gynaecologists (RCOG).

### Search strategy

An initial search strategy was designed a priori as part of the protocol development. We undertook various basic, restricted searches in Medline and Google Scholar to uncover some articles relevant to the review topic. Based on these searches, we conducted an analysis of the text words in the titles and abstracts and the index/medical subject heading (MeSH) terms used to describe each article to inform the development of an initial comprehensive search strategy. We also reviewed published search strategies from reviews on similar topics to harvest keywords related to our review question.

The initial search strategy was developed in Medline via the EBSCO platform based on the Sample and Phenomenon of Interest components of the SPIDER framework described above and was refined through team discussion and piloting to ensure feasibility. The Medline strategy (which can also be run in CINAHL Ultimate and APA PsycInfo via the EBSCO platform) was subsequently adapted for Embase and Scopus. For example, for Embase, MeSH terms were replaced with equivalent Emtree terms, while Scopus requires simpler keyword-based queries rather than complex syntax. The search strategies were independently reviewed and further refined by two experienced medical subject librarians. The search strategy for Medline is provided in box 1.

Box 1 **Medline search strategy**

((((MH ‘Endometriosis’ OR MH ‘Adenomyosis’ OR MH ‘Dysmenorrhea’ OR MH ‘Pelvic pain’)) OR ((XB (endometri* OR adenomyosis OR dysmenorrh* OR (painful adj3 menstruation) OR (painful adj3 period*) OR (menstrual adj3 disorder*) OR (period adj3 pain) OR (menstrual adj3 health) OR (menstrual adj3 pain) OR (menstrual adj3 cramp*) OR ‘chronic pelvic pain’))))) AND ((XB (((contrast or hot or heat or cold) adj3 therap*) or ‘plunge pool’ or ‘cold water immersion’ or ‘hot water immersion’ or ‘contrast water immersion’ or ‘contrast bath’ or ‘ice bath’ or ‘contrast water therap*’ or thermotherap* or ‘thermal therap*’ or ‘thermal shock’ or cryotherap* or cryothermal or hydrotherap* or sauna or ‘infrared sauna’ or ‘contrast shower’ or ‘therap* hyperthermia’ or ‘infrared therap*’)))

We will search the Title and Abstract fields in Medline, and the Title Abstract or Keyword fields in Scopus. In Embase, to increase the precision of the search, we will limit results to articles where the specific Emtree terms are the focus of the article. Searches will be further filtered to exclude studies focused on men and non-human studies. The initial search strategies may be refined as the review progresses and following PPI input.

### Data management

The lead author (LEC) will undertake and manage all searches given their extensive experience conducting systematic and scoping reviews. All records identified by the database searches will be imported into the reference management software programme EndNote 2025 and duplicates removed. The remaining records will be imported into the review management software programme Covidence for screening. Any eligible records identified from citation searching that do not appear in the database search will be added to Covidence manually. Included studies will be saved as PDF files in a shared folder and the citations managed in EndNote 2025. Data from included studies will be extracted within Covidence and downloaded to a Microsoft Excel spreadsheet.

### Study selection

An abstract screening tool was developed to assist with the initial screening process (online supplemental appendix 2). To ensure high inter-rater reliability, a calibration exercise will be conducted at the beginning of the screening process. For calibration, a random sample of 30 titles and abstracts will be screened independently in duplicate by two reviewers (RA and LEC) against the predefined inclusion criteria and in combination with the screening tool.^37^ Any discrepancies will be discussed with a third reviewer (MB) and amendments made to the screening tool if necessary. Following calibration, title and abstract screening will be conducted independently by two reviewers. The reviewers will categorise the articles as ‘Yes’, ‘No’ or ‘Maybe’; all ‘Yes’ and ‘Maybe’ articles will be selected and retrieved for full-text screening.

Subsequently, a second calibration exercise will be conducted. For this exercise, 10% of full texts will be screened independently in duplicate against the inclusion criteria by two reviewers (RA, LEC). Any discrepancies will again be discussed and resolved with a third reviewer (MB). Following calibration, full texts of potentially eligible studies will be assessed for inclusion independently by two reviewers. The reviewers will categorise the articles as either ‘Yes’ or ‘No’. All studies excluded at the full-text screening stage will be reported with reasons for exclusion provided. At both stages of the screening process (abstract and full-text screening), any discrepancies will be resolved through team discussion or a third reviewer.

### Data extraction

A wide range of study designs will be eligible for inclusion. To manage this, the review will follow the principles of mixed-methods framework synthesis.^38^ Specifically, data will be extracted, mapped and charted according to the review questions. We will develop a-priori a standardised data extraction form within Covidence. The form will be piloted on two studies before use to ensure that all relevant data are captured and will be modified as necessary. It will then be refined and updated as needed as extraction progresses and in accordance with PPI input. Data from each included study will be extracted by two reviewers independently and discrepancies will be resolved by a third reviewer. Supplementary information will be obtained, when necessary, by contacting the authors of the primary studies.

### Data items

We will extract data on the article information, methods, sample characteristics, intervention type, outcomes and key findings (table 2). Additional relevant data items may be identified as the review progresses.

**Table 2:** Data items to be extracted from included studies

Category	Data to be extracted
Article information	Authors, title, journal, year of publication, country.
Methods	Research type (quantitative, qualitative, mixed methods), study design, research question(s), study aims and objectives, data sources.
Sample characteristics	Sample size, age, condition(s), gender, ethnicity, socio-demographics.
Intervention type	Type of therapy (hot, cold, contrast), specific intervention/exposure, duration of intervention, setting, comparator and number of groups if applicable.
Outcomes	Outcomes assessed, details of instrument(s) used to measure outcomes.
Key findings	Any results that relate to the review question. Estimates of effect, eg, p values, odds/HRs, CIs.

### Synthesis and presentation of results

As the aim of a scoping review is to provide an overview of the extent and nature of a body of literature, data will be synthesised using framework synthesis (table 3). The framework will be further developed as the review progresses. Findings will be presented descriptively in tabular and narrative format. We will include a PRISMA flowchart in the results section of the review publication to report the study selection process. We will provide frequency counts of the amount, type and distribution of the studies included in the review and summarise the number of studies examining specific populations, interventions and outcomes. This will assist in highlighting where additional research may be required to fill evidence gaps.

**Table 3:** Initial synthesis framework

Section	Key questions	Sub-component
Evidence landscape (contextualisation)	What literature exists and what are its characteristics?	Study title/aims
		Study design
		Location/country
		Publication context
Population & intervention characteristics	Who was studied, and how was the therapy delivered?	Condition focus
		Population descriptors
		Thermal modality
		Delivery parameters
		Setting & design
Outcome domains—pain	What pain outcomes were measured and what was reported?	Pain severity
		Pelvic/chronic pain
		Analgesic use (any other therapies used?)
		Proposed mechanism for pain relief
Outcome domains—physical well-being	What physical well-being outcomes were measured and what was reported?	Fatigue
		Sleep quality
		Bloating/gastrointestinal symptoms
		Bladder symptoms
		Physical functioning/activity
Outcome domains—mental well-being & QoL	What mental well-being/QoL outcomes were measured and what was reported?	Anxiety
		Depression/mood
		Quality of life (QoL)
		Patient experience (qualitative)
Charting & gap mapping	What patterns, convergences and gaps emerge across the evidence?	Areas of convergence
		Areas of divergence/inconsistency
		Methodological observations
		Evidence gaps identified
		Future research priorities
		Clinical/practice implications

## Ethics and dissemination

The proposed study does not require ethical approval as it is a scoping review of the existing literature and will only describe and synthesise previously published data. Throughout data extraction and analysis, we will consult with the EMPOWER-CONNECT advisory board and the EMPOWER-VOICES PPI panel who will provide insight and feedback on the study findings, assist in disseminating the findings, and contribute to future research proposals. We will produce and disseminate creative outputs (eg, visual illustrations) that capture discussions during the PPI workshop. These outputs will be coproduced and designed to complement traditional research dissemination. We plan to disseminate the review findings through publication in a peer-reviewed journal, conference presentations, policy summaries and posts on professional social media in lay language, and webinars with relevant groups and networks. Key networks include the Social Sciences in Endometriosis (SEEN) network, the Primary care Endometriosis and Adenomyosis Research and Learning (PEARL) network and the 4M (Menarche, Menstruation, Menopause and Mental Health) consortium. In addition, we will use the findings to inform a planned feasibility study on the use of contrast therapy to support symptom management and well-being among those with endometriosis, and to inform additional research projects to address any evidence gaps identified by the review.

## Supplementary material

10.1136/bmjopen-2026-120614 online supplemental appendix 1

10.1136/bmjopen-2026-120614 online supplemental appendix 2

## Data Availability

No data are available.
