# Supplementary material for: Whole-body thermal therapies for symptom management and well-being in people assigned female at birth with chronic pelvic pain, dysmenorrhoea, endometriosis or adenomyosis: a scoping review protocol
Source: BMJ Open. 2026 Aug 3;16(8):e120614. doi: 10.1136/bmjopen-2026-120614 (PMC13436099; doi:10.1136/bmjopen-2026-120614)
Supplement: Supplementary file 1 [file bmjopen-2026-120614.reviewer_comments.pdf]

PEER REVIEW HISTORY

BMJ Open publishes all reviews undertaken for accepted manuscripts. Reviewers are asked to complete a checklist review form and are provided with free text boxes to elaborate on their assessment. These free text comments are reproduced below.

ARTICLE DETAILS

Title (Provisional)

Whole-body thermal therapies for symptom management and wellbeing in people assigned female at birth with chronic pelvic pain, dysmenorrhea, endometriosis, or adenomyosis: a scoping review protocol

Authors

Cowley, Laura Elizabeth; Bianchim, Mayara S; Isherwood, Kate; Roberts, Judith; Rees, April; Joseph, Rachel; Anthony, Rebecca

---

VERSION 1 - REVIEW

**Reviewer**	**1**
**Name**	**Donato, Helena**
**Affiliation**	**Centro Hospitalar e Universitario de Coimbra EPE, Documentation and Scientific Information Service**
**Date**	**17-Jun-2026**
**COI**	**None**

---

1. I think the title could be improved. I suggest: Whole-body thermal therapies for symptom management and wellbeing in people assigned female at birth with chronic pelvic pain, dysmenorrhea, endometriosis, or adenomyosis: a scoping review protocol

2. The authors state that they searched the Cochrane Database of Systematic Reviews and PROSPERO to identify existing systematic or scoping reviews. However, scoping reviews are neither registered in PROSPERO nor published in the Cochrane Database. How did the authors ascertain that there are no published or ongoing scoping reviews on this topic?

3. The Methods section is predominantly written in the past tense, suggesting that several methodological procedures may already have been undertaken. This raises a broader concern regarding the purpose of a review protocol. Protocols are intended to prospectively specify the review methods before the review is conducted, thereby enhancing transparency and reducing the risk that methodological decisions are influenced by emerging findings. Apart from preliminary searches performed to establish the need for the review, most methodological steps would not normally have been completed at the protocol stage. The current wording gives the impression that the protocol may have been developed after key

methodological decisions had already been implemented. The authors should clarify which activities have already been undertaken and revise the manuscript to better reflect the prospective nature and rationale of a review protocol.

4. It was not clear to me why the year 2000 was selected as the start date for the search. What was the rationale for this choice?

5. Google Scholar is known to index content from a wide range of sources, including journals that may be considered predatory. Therefore, its use as a source of grey literature raises concerns regarding the quality and credibility of the records retrieved. The authors should clarify how they intend to assess the legitimacy of sources identified through Google Scholar and what criteria will be used to distinguish potentially predatory publications from credible sources.

6. The authors may wish to consider searching the Web of Science Core Collection. In addition to serving as a major bibliographic database, it facilitates citation tracking, allowing both backward and forward citation searching. This approach can enhance the comprehensiveness of the review by identifying potentially relevant studies that may not be retrieved through the predefined search strategy alone.

7. The reference list would benefit from further editorial revision. The bibliographic references are not consistently formatted, and some appear to be missing key elements. Greater attention to accuracy, completeness, and consistency in the reference list is needed to ensure that all sources can be clearly identified and traced

---

VERSION 1 - AUTHOR RESPONSE

**Reviewer comments**	**Response**
1. I think the title could be improved. I suggest: Whole-body thermal therapies for symptom management and wellbeing in people assigned female at birth with chronic pelvic pain, dysmenorrhea, endometriosis, or adenomyosis: a scoping review protocol	Thank you for this suggestion, we have now amended the title accordingly.
2. The authors state that they searched the Cochrane Database of Systematic Reviews and PROSPERO to identify existing systematic or scoping reviews. However, scoping reviews are neither registered in PROSPERO nor published in the Cochrane Database. How did the authors ascertain that there are no published or ongoing scoping reviews on this topic?	Thank you for noticing this, the Cochrane Database and PROSPERO were only searched for published or ongoing systematic reviews (not scoping reviews). This has now been amended in the manuscript (page 5).
3. The Methods section is predominantly written in the past tense, suggesting that several methodological procedures may already have been undertaken. This raises a broader concern regarding the purpose of a	Thank you for highlighting this. To clarify, we have written part of the “search strategy” section in the past tense because we designed the initial search strategy as part of the protocol development. This is in-keeping

review protocol. Protocols are intended to prospectively specify the review methods before the review is conducted, thereby enhancing transparency and reducing the risk that methodological decisions are influenced by emerging findings. Apart from preliminary searches performed to establish the need for the review, most methodological steps would not normally have been completed at the protocol stage. The current wording gives the impression that the protocol may have been developed after key methodological decisions had already been implemented. The authors should clarify which activities have already been undertaken and revise the manuscript to better reflect the prospective nature and rationale of a review protocol.	with the nine-step process for conducting scoping reviews described in the guidance authored by Peters et al (2020). In this guidance, the first three steps are undertaken during protocol development, including “describing the planned approach to evidence searching”. We explained that the search strategy may be refined as the review progresses. We have now added a sentence at the beginning of the search strategy section on page 9 to clarify that an initial search strategy was designed as part of protocol development. In line with this, we also developed a-priori an abstract screening tool (first sentence of the study selection section), which is why this sentence is also written in the past tense.<br><br>We can confirm that, crucially, the review did not begin, that is, none of the searches had been carried out, until after the protocol had been fully developed, signed off by all team members and submitted for publication.<br><br>We have now double checked the rest of the methods section to ensure that the tenses are correct and can confirm that everything else is written in the future tense, as appropriate.
4. It was not clear to me why the year 2000 was selected as the start date for the search. What was the rationale for this choice?	We explained the rationale for selecting the year 2000 as the start date for the search in the Eligibility section of the methods (pages 7-8), as follows:<br>“The search will be restricted to studies published from 2000 to 2026 to enhance the feasibility of the review and ensure relevance to contemporary clinical practice and research standards. Diagnostic approaches, management pathways, and delivery of non-pharmacological therapies for endometriosis and related conditions have evolved substantially over the past two decades.[34] Additionally, studies published after 2000 are more likely to employ standardised outcome measures and reporting frameworks, improving comparability and interpretability of findings.”
5. Google Scholar is known to index content from a wide range of sources, including journals that may be considered predatory. Therefore, its use as a source of grey	Thank you for pointing this out, however we will not use Google Scholar to search for grey literature. We only used Google Scholar to identify some initial articles relevant to the

literature raises concerns regarding the quality and credibility of the records retrieved. The authors should clarify how they intend to assess the legitimacy of sources identified through Google Scholar and what criteria will be used to distinguish potentially predatory publications from credible sources.	review topic, to assist in the development of the search strategy.<br><br>In the eligibility criteria section on Page 7, we specified that grey literature will be excluded. However, we have now explained in the Information Sources section on Page 9 that we will search the following grey literature sources: World Health Organisation, United Nations Children's Fund (UNICEF), United Nations Population Fund (UNFPA), National Institute for Clinical Excellence (NICE), Endometriosis UK, Welsh Government, Welsh Parliament (Senedd), NHS Wales, NHS England, the Menstrual Health Project, Wellbeing of Women, the Endometriosis Foundation, European Society of Human Reproduction and Embryology (ESHRE) and The Royal College of Obstetricians and Gynaecologists (RCOG).
6. The authors may wish to consider searching the Web of Science Core Collection. In addition to serving as a major bibliographic database, it facilitates citation tracking, allowing both backward and forward citation searching. This approach can enhance the comprehensiveness of the review by identifying potentially relevant studies that may not be retrieved through the predefined search strategy alone.	Thank you for this suggestion, we will search the Web of Science database alongside the others we have mentioned. We have added this database to the Information Sources section (page 9).
7. The reference list would benefit from further editorial revision. The bibliographic references are not consistently formatted, and some appear to be missing key elements. Greater attention to accuracy, completeness, and consistency in the reference list is needed to ensure that all sources can be clearly identified and traced	Thank you for noticing this, we have now double checked all the references and reformatted many of them to ensure consistency and completeness.
