# Supplementary material for: Whole-body thermal therapies for symptom management and well-being in people assigned female at birth with chronic pelvic pain, dysmenorrhoea, endometriosis or adenomyosis: a scoping review protocol
Source: BMJ Open. 2026 Aug 3;16(8):e120614. doi: 10.1136/bmjopen-2026-120614 (PMC13436099; doi:10.1136/bmjopen-2026-120614)
Supplement: Supplementary file 3 [file bmjopen-16-8-s002.docx]

**Supplementary Appendix 2: Abstract screening tool**

**Review title**

The use of whole-body thermal therapies to support symptom management and wellbeing in people assigned female at birth with dysmenorrhea, pelvic pain, endometriosis, or adenomyosis

**Citation, Title, and Abstract Screening**

*This screening tool should be used alongside the inclusion/exclusion criteria in the protocol paper*

1. Does the **citation** indicate publication on or after the year 2000?

a. Yes: continue screening

b. No: stop screening and exclude

2. Does the **title or abstract** use English?

a. Yes: continue screening

b. No: stop screening and exclude

**Abstract Screening**

3. Does the **abstract** indicate that the study is an empirical study including Randomised controlled trials, quasi-experimental studies, focus groups, intervention studies, interview studies, survey studies, case studies, observational studies, or clinical guidelines if they provide information or evidence about whole-body thermal therapies (e.g. quantitative, qualitative, or mixed methods)

a. Yes or Unsure/Unclear: continue screening

-Key words: regression, covariate, modeling, correlation, variance, ethnography, discourse analysis, thematic analysis, interpretative phenomenological analysis, content analysis, grounded theory, interviews, inquiry, trial, and mixed

b. No: stop screening and exclude

-For example: letters, commentaries, editorials, reviews, book chapters, study protocols

4. Does the **abstract** indicate that the study is published in a peer-reviewed journal?

a. Yes or Unsure/Unclear: continue screening

b. No: stop screening and exclude

-For example: master’s and PhD dissertations/theses, conference proceedings and abstracts

5. Does the **abstract** indicate that the sample are women or people assigned female at birth?

a. Yes or Unsure/Unclear: continue screening

b. No: stop screening and exclude

6. Does the **abstract** indicate that the sample includes participants with dysmenorrhea, suspected or confirmed endometriosis, adenomyosis, or unexplained chronic pelvic pain?

a. Yes or Unsure/Unclear: continue screening

b. No: stop screening and exclude

7. Does the **abstract** indicate that the use or effect of whole-body thermal therapies was studied?

a. Yes or Unsure/Unclear: continue screening

-Key words: whole-body heat therapy, whole-body cold therapy, and contrast therapy, delivered through modalities such as baths, immersion, showers, or saunas.

b. No: stop screening and exclude

8. Does the **abstract** indicate that outcomes include symptom management and/or wellbeing?

a. Yes or Unsure/Unclear: continue screening

-Key words: pain, wellbeing, inflammation, symptom, self-care, quality of life

b. No: stop screening and exclude

**Decision**: **Should this article be included for full-text review?**

a. **Yes**, all 8 screening questions answered Yes or Unclear

b. **No**, at least one answers “No”
